# Respirator-integrated continuous gas sampling for broadband optical spectroscopy

**DOI:** 10.1038/s41598-026-69327-3

**Published:** 2026-09-04

**Authors:** Aleksandra Borek-Dorosz, Jan Ornik, Marco Personeni, Stefan Haering, Maximilian Högner, Felix Paries, Margit Leitner, Jens-Uwe Weiß, Ignacio Rubio, Michael Bauer, Ioachim Pupeza

**Affiliations:** 1 Leibniz Institute of Photonic Technology ─ Member of the Research Alliance, Leibniz Health Technologies, 07745 Jena, Germany; 2 Cluster of Excellence Balance of the Microverse, Friedrich Schiller University Jena, 07743 Jena, Germany; 3 Physics Department and State Research Center OPTIMAS, University of Kaiserslautern-Landau, 67663 Kaiserslautern, Germany; 4 Fraunhofer Institute for Industrial Mathematics ITWM, 67663 Kaiserslautern, Germany; 5 Department of Anesthesiology and Intensive Care, Leibniz Center for Photonics in Infection Research (LPI), Jena University Hospital, Friedrich Schiller University, Am Klinikum 1, 07749 Jena, Germany; 6 Center for Sepsis Control and Care (CSCC), Jena University Hospital, Am Klinikum 1, 07749 Jena, Germany

**Keywords:** Breathomics, FTIR spectroscopy, Respiratory gas monitoring, Automated breath collection, Biological techniques, Chemistry, Optics and photonics, Physics

## Abstract

With its high molecular complexity, human breath lends itself as an information-rich sample for non-invasive biomedical diagnostics. Among emerging multi-variate analytical techniques for human breath, laser-based optical spectroscopy constitutes a particularly promising novel combination of short measurement times and high concentration dynamic range for molecular detection. Here, we present an automated system designed for continuous collection of exhaled breath directly from a respirator, and subsequent spectroscopic analysis. By obviating any additional intermediate sample storage or transport, our method minimizes setup complexity and sample contamination, while avoiding interference with the operation of the respirator. In this proof-of-principle study, we present automated and quantitative monitoring of three substances introduced in an artificial test lung ventilated by the respirator. Employing a standard Fourier-transform infrared spectrometer for molecular quantification, individual measurements were performed every 7 min over 24 h. The stability and analytical capability of the breath sampling system presented here, along with improvements in terms of spectral resolution and detection dynamic range obtainable through state-of-the-art laser-based spectroscopy promise fast and quantitative monitoring of the human breath volatilome. Coupled to state-of-the-art spectroscopy this may pave the way to monitor metabolites in real time in critically ill patients over the entire physiologically relevant molecular concentration range.

**Supplementary Information:**

The online version contains supplementary material available at https://doi.org/10.1038/s41598-026-69327-3.

## Introduction

Human breath analysis offers significant diagnostic potential, as it enables non-invasive sample collection and analysis. Breathomics, a relatively new research field, involves the analysis of volatile organic compounds (VOCs) in exhaled breath to monitor health and disease states^1^. By capturing and profiling the unique “breathprint” of VOCs, this approach provides insights into pulmonary and systemic, in particular metabolic physiological processes^2,3^. This might allow a repeatable and patient-friendly alternative to conventional diagnostics^4,5^. Exhaled breath is a complex biological matrix containing proteins^6^, drug metabolites^7^, and around 4,000 known VOCs^8^. Notably, VOC concentrations span a wide dynamic range, from highly abundant gases like carbon dioxide, oxygen or water vapour at percentage levels (%) to trace biomarkers in lower concentrations such as isoprene, acetone^9^, hydrocarbons (detectable at parts-per-billion [ppb] levels) butanal, and hexanal (at parts-per-trillion [ppt] levels)^10,11^.

The VOCs present in exhaled breath are closely linked to physiological and pathological processes^12^, reflecting underlying biochemical changes in metabolism^13^. Alterations in VOC profiles have already been associated with diverse conditions, including infectious diseases^14^, cancers^15,16^, metabolic disorders^17^, and inflammation^18^. Such changes arise from shifts in endogenous metabolism or microbiome activity^13^, as well as exogenous exposures^13^, positioning breath VOCs as sensitive biomarkers of health and disease. Consequently, breathomics holds substantial promise for early diagnosis, disease monitoring, and personalized medicine^19,20^.

For instance, shifts in VOC profiles occur in respiratory conditions like asthma^21^, chronic obstructive pulmonary disease^22^, or interstitial lung diseases^21^, where compounds such as p-xylene^22^, acetoin^21^, and ethylbenzene^21^ are elevated due to altered oxidative stress or lipid peroxidation. Metabolic disorders, cancers, and infections similarly modulate specific VOCs; elevated acetone levels in diabetes^23^, whereas hydrocarbons may indicate oxidative damage in tumors^10^. These changes arise from endogenous pathways (e.g., glucose metabolism yielding ethanol)^24^ or microbial interactions in the airways^25^. In critically ill patients on mechanical ventilation, real-time VOC monitoring is crucial, particularly for detecting sepsis^26^— a life-threatening condition driven by dysregulated host responses to infection that remains challenging to treat due to delayed diagnosis and mortality rates exceeding 20–50%^27,28^. Current techniques like blood cultures are slow, taking hours to days, they lack sensitivity for early-stage diagnosis of sepsis^29,30^ and are associated with complications due to invasive sampling^31^. As such, the unmet need for real-time monitoring and diagnostics at the molecular level, as well as their standardization, persists^32^.

This vast dynamic range of VOCs, while analytically challenging due to the need for sensitive detection techniques like gas chromatography-mass spectrometry (GC-MS)^33^ or ion mobility spectrometry^34^, underscores the richness of breath as a metabolic window (a time-resolved, non-invasive window for tracking metabolic change). In this area of research, spectroscopic methods have already been applied. These methods, especially Raman^35^ and infrared (IR)^36^ spectroscopy techniques, are emerging as powerful tools for non-invasive, label-free breath analysis, enabling detection of disease-related VOCs. Spontaneous Raman spectroscopy in exhaled breath can identify carbon dioxide, methane, and specific VOCs in ppm levels^35^. Surface-enhanced Raman spectroscopy (SERS) with plasmonic substrates (e.g., Au-decorated nanowire networks), has achieved detection limits down to low-ppb levels for model analytes and allows for discrimination between healthy and diseased subjects (e.g., upper respiratory tract infections, lung and gastric cancer, diabetes)^37,38^. Mid- and near-IR spectroscopy exploits molecular vibrations in the C–H, N–H, O–H, and C = O regions, enabling quantification of major breath components such as carbon dioxide, carbon monoxide, acetone, and other VOCs^39,40^.

Standardisation of breath collection remains crucial for minimising contaminants and ensuring reproducibility. Existing studies use several methods for breath sample collection, each with specific advantages and limitations. One example are mouthpiece connected to Tedlar bags^41^, which enable short-term storage and subsequent analysis. These bags, however, may introduce background contamination or partial sample loss due to adsorption of VOC^42^. The breath collected in such ways can be subsequently studied with GC-MS^43^, e-nose devices (electronic nose)^43^ or Fourier-transform infrared (FTIR) spectroscopy^44^. Another sampling approach uses special syringes near the mouth of the breathing patient^44^, however with this method ambient air around the mouth can also be captured. There are also different nasal or oral masks, like ReCIVA^45,46^ which enable the connection directly to adsorbent tubes, and subsequent GC-MS measurement^41^. Mouth pieces with tube connections to Tedlar bags for intermediate sample storage have also been used^47–49^. When it comes to the collection of breath from the respirator, the exhaled air is most commonly collected from the ventilator exhaust or expiratory outlet into a Tedlar bag^50^ or withdrawn with a syringe^50–52^. In practice, the choice of the collection method depends on the analytical platform, the target analytes, and the need to balance sample integrity, stability, and reproducibility^32^. For critically ill patients who require mechanical ventilation, adjustments in the breath sampling procedure are required. For example, Tedlar bags have been connected to the exhaust of a respirator to sample the exhaled air and analyse its VOC content^53,54^, and also syringes were used to withdraw breath from within the respirator before the heat and moisture exchanger (HME) filter^50^. On the contrary our approach aims to enable application of VOC monitoring in real-time and for ventilated patients, i.e. a high-risk population of patients where diagnostics of e.g., infectious complications or metabolic derangement is time critical and difficult with currently available techniques.

Here, we present a technical implementation of a gas sampling unit for the automated extraction of exhaled breath from a respirator for direct and continuous spectroscopic analysis. We use a medically approved pump for anaesthetic gas monitoring (Vamos, Dräger) and a set of automated valves to sample the ventilated air from the test lungs to a single-pass, custom-made measurement cell placed in a commercial FTIR spectrometer for subsequent spectroscopic measurements. In this proof-of-principle study, pure synthetic gases diluted in nitrogen were first measured and analysed to establish the standard operating protocol and determine limits of the detection (LOD) and quantification (LOQ). Furthermore, by monitoring the presence of chosen synthetic gases in the ventilated test lung over an entire day, we demonstrate the applicability of the gas sampling unit for future continuous monitoring of exhaled breath with broadband spectroscopic techniques in ventilated patients. With the recent advent of infrared spectroscopic techniques drawing from the outstanding coherence properties of modern femtosecond lasers to cover the entire physiologically relevant molecular concentration range, this paves the way to new paradigms in precision intensive-care medicine^55,56^.

## Materials and methods

### Materials

Acetone (CAS: 67-64-1), pentane (CAS: 109-66-0) and methanol (CAS: 67-56-1) were acquired from Merck in liquid forms. The purity of these chemicals was in the range of 97%–99.9%.

### Preparation and handling procedure of synthetic gas samples

Synthetic gas samples containing only a single molecular species of interest (i.e., VOC) were prepared by injecting a predetermined amount of liquid sample with a syringe (a gas-tight Hamilton syringe) through a septum into an evacuated stock bulb with a volume of 1,000 cm^3^, where the liquid sample evaporated completely (stock bulb, Figure S1, in the supplementary information). Subsequently, nitrogen, an IR-inactive gas, was filled into the stock bulb until the pressure in the stock bulb reached ambient pressure. This was achieved by connecting a Tedlar bag filled with nitrogen to the stock bulb and opening the connecting valve for a short period of time. The concentration of the synthetic gas depends on the amount of introduced liquid sample and was calculated by assuming ideal gas conditions (Equations S1-S3 in the SI). To achieve lower concentrations (< 1,000 ppm) an additional dilution step was required (sample bulb, Figure S1). The diluted samples were prepared by taking a defined amount of gaseous sample from the stock bulb through a septum with a gas-tight syringe and injecting it into another evacuated gas bulb (i.e., sample bulb) with a volume of 1,000 cm^3^ (Equations S4-S5 in the SI). This was followed by connecting a Tedlar bag filled with nitrogen to the sample bulb and opening the valve for a short period until the pressure equalled ambient pressure. The volumes used for the preparation of stock samples and in the dilution steps are provided in Table S2 in the supplementary information. The prepared concentrations were calculated based on the assumption of ideal gas behaviour (see supplementary information) and should be treated as approximate values.

After the sample with the desired concentration was prepared, the tube segments connecting the sample bulb with the measurement cell were evacuated, and then the valves closing the measurement cell and sample bulb were opened. In this way, the prepared gas was allowed to flow from the sample bulb into the measurement cell, ensuring a well-defined sample composition at a pressure of 920 mbar in the measurement cell. For reference, the measurement cell was filled with nitrogen with the use of Tedlar bag. Between sample preparations, syringes, bulbs, and measurement cell underwent cleaning cycles, consisting of evacuation and flushing with dry air and nitrogen. The cleaning procedures are described in M.1 in the SI. All samples were prepared and measured five times.

### Continuous gas sampling experiments from the respirator

**Fig. 1:**
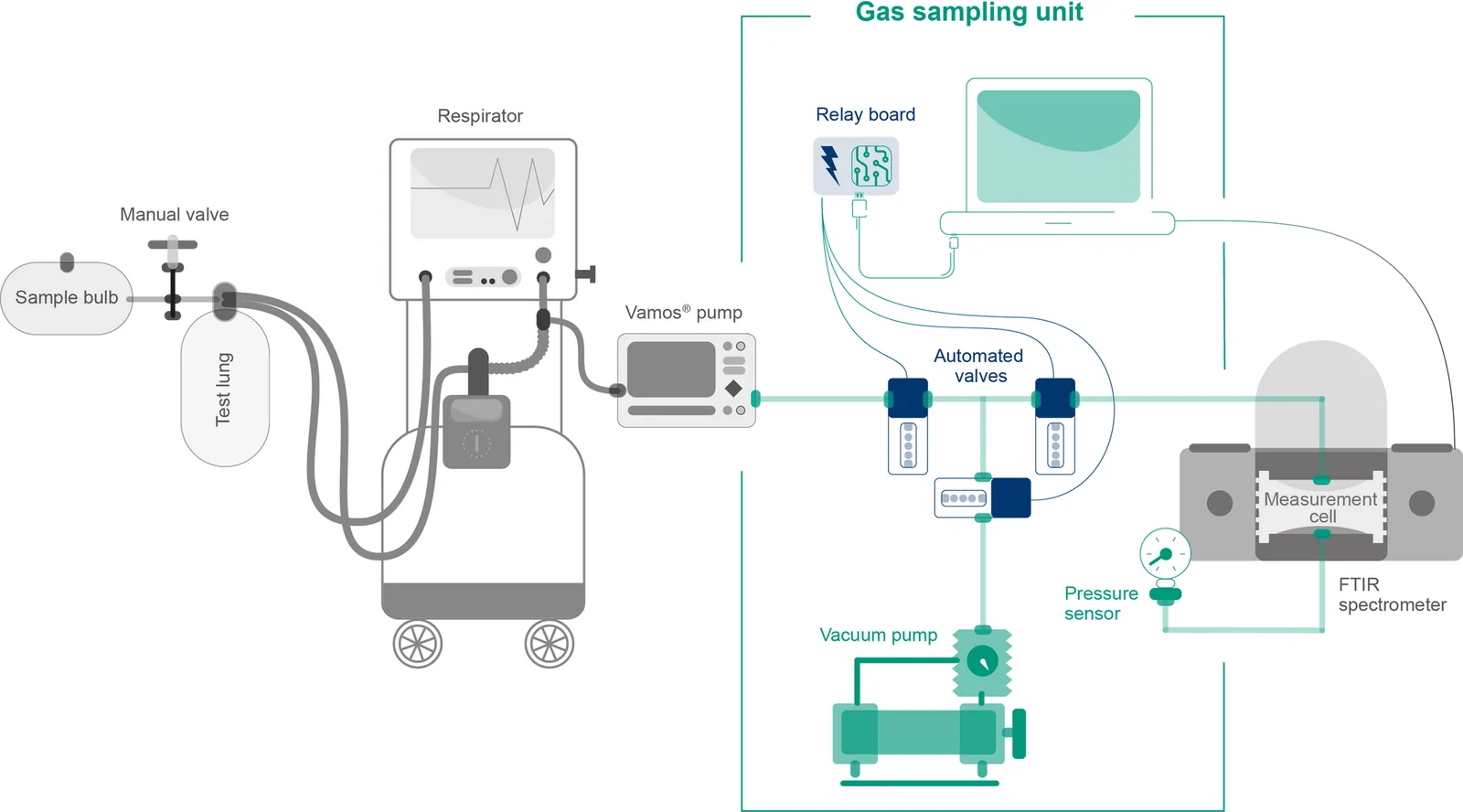
Schematic representation of sample collection and measurements with connection to respirator. The sample bulb and measurement setup are connected to the respirator near the ventilated test lung, enabling the circulation of the prepared gases inside respirator environment (left). The Vamos pump is connected to the respiratory exhaust and further linked to the measurement cell via the gas sampling unit, which consists of automated valves, vacuum pump, pressure sensor, relay board and computer (middle). The measurement cell was located inside the FTIR spectrometer and was connected to the pressure sensor (right).

The experimental setup for the continuous gas sampling from the respirator is shown in Fig. 1. The setup consists of three main parts: (1) respirator (Evita, Dräger), test lung (Dräger SelfTestLung™), sample bulb and Vamos pump (Dräger), (2) gas sampling unit consisting of three computer-controlled automated valves, pressure sensor and vacuum pump, and (3) FTIR spectrometer (Bruker Vertex 70) with measurement gas cell. During the continuous 24-hour experiments, the respirator was connected to the test lung and operated in the volume control – mandatory minute volume (VC-MMV) mode (see section M3 in the SI for details). The Vamos pump was connected at the exhalation line of the respiratory circuit where it sampled a part of the exhaled air at a constant rate of 150 ml/min. The sampled gas was processed by the gas sampling unit either by guiding it into the evacuated measurement cell for subsequent spectroscopic analysis or guiding it into the surrounding.

The automated gas sampling unit represents the central part of the experimental setup enabling continuous gas sampling with subsequent spectroscopic analysis. The three computer-controlled valves (MHE3-M1H-3/2G-1/8-K, Festo) can be configured such that the measurement cell is either connected to the vacuum pump (HiScroll 12, Pfeiffer Vacuum) during the evacuation step or to the Vamos pump for the sample loading or closed to keep the conditions during the spectroscopic measurements constant. The valves were controlled via custom Python (v3.13) control software with a relay board (USB-RELAY-8_A, Deditec) powered by a Voltcraft ESP-3005 S power supply and connected to a computer via USB (PySerial). The pressure sensor (Pirani PCR280, Pfeiffer Vacuum) was installed to the measurement cell and interfaced with the control software via a USB-to-RS485 adapter using the PySerial (v3.5) package. To minimise leakage and interference from ambient air, the primary ports of the valves were sealed, except for the valve upstream of the Vamos pump. A secondary opening in this valve ensured undisturbed operation of the Vamos pump and consequently undisturbed respirator operation. Valve switching was controlled based on real-time pressure thresholds and the operational state of the FTIR spectrometer. The Bruker Vertex 70 spectrometer was operated through the OpusPythonAPI (v1.0.0) software, enabling command triggering and synchronous data acquisition by requests to the HTTPS-based communication.

For the two 24-hour experiments, the ventilated pathways including the test lung were spiked with the mixtures of previously studied species (i.e., acetone, methanol, pentane). For the first experiment 5 ml of liquid acetone and methanol (binary mixture) were interchangeably injected into the initially evacuated sample bulb until 65 ml of each species were introduced. For the second experiment, 65 ml of each of the three liquids (acetone, methanol and pentane - ternary mixture) were injected in the same way into the sample bulb. After repeating the evacuation of the tubing and measurement cell to 1.5 mbar and flushing them with nitrogen to 0.95 bar three times, the experiment was started by repeating the measurement cycle every seven minutes.

For a measurement cycle resulting in a recorded absorbance spectrum the following procedure was executed by the gas sampling unit: (1) Evacuate the measurement cell down to 1.5 mbar; (2) Close all valves isolating the measurement cell; (3) Trigger a reference measurement of the FTIR and wait for completion; (4) Evacuate the measurement cell down to 1.5 mbar; (5) Open sample inflow from the respirator to the measurement cell up to 0.95 bar; (6) Close all valves, isolating the measurement cell; (7) Trigger a sample measurement of the FTIR and wait for completion; (8) Evacuate the measurement cell, wait for a timer that triggers the next cycle and datapoint acquisition. One hour after the experiment was started, the valve between the sample bulb and the ventilated pathways was opened, allowing the vapour of the studied species to spike the air in the test lung.

### FTIR measurements

All spectra were collected using an FTIR spectrometer (Vertex 70, Bruker Optics, Germany). The spectrometer was equipped with a mid-infrared source and an RT-DLa-TGS (room-temperature deuterated L-alanine-doped triglycine sulphate) detector and covered the spectral range of 500–4000 cm^− 1^. All spectra were collected with a spectral resolution of 1 cm^− 1^ and 100 scans (for single molecular species) or 50 scans (for continuous gas measurements) per spectrum. The spectrometer was continuously purged with dry air to reduce water vapour contribution from the instrument. The reference spectrum was recorded immediately before each sample measurement. All samples were measured in a metallic, single-pass, 14.3-cm long measurement cell with a volume of 50 cm^3^ and IR-transparent ZnSe windows.

### Data analysis

Spectra were individually baseline-corrected in their full range by subtracting a second-order polynomial fitted to four spectral regions (775–860, 2030–2200, 2540–2560, 3255–3425 cm^− 1^) devoid of absorption bands from the analytes, water vapour and carbon dioxide. Further analysis focused exclusively on spectral regions containing the distinct spectral features of the studied species, i.e. 865–1285 and 2560–3250 cm^− 1^, and thereby also excluding regions in which water vapour and CO_2_ are strongly absorbing.

Data analysis focused on the concentration retrieval of individual species in the ternary gas mixture. Partial least square regression (PLSR) has proved to be a robust analytical tool to evaluate absorbance gas spectra with strongly overlapping features^57–59^ and is therefore the elected method for the concentration retrieval of continuous measurements involving the respirator.

Prior to model development, single-species spectral data were curated by excluding spectra recorded at concentration levels that did not permit unambiguous species identification. To determine the concentration range suitable for model training, separate PLSR models were constructed for each species using concentration-averaged spectra and their corresponding nominal concentrations. Given the one-dimensional chemical variability of each dataset, a single latent variable (LV) was used in each model. The model performance was evaluated using leave-one-group-out cross-validation (LOGO-CV)^60^, where all spectra acquired at a given concentration level were omitted from the training set and used for validation. The resulting calibration curves were used to determine the limit of detection (LOD) and limit of quantification (LOQ) for each species. Both quantities were calculated according to the DIN 32,645 standard at a confidence level of 98%^61^. The LOQ, being the more stringent criterion, was subsequently used as the concentration threshold for spectrum inclusion in the multi-species analysis.

In order to establish a correlation between the prepared concentration and the characteristic absorbance intensity of each species in the ternary mixture, a three-components PLS model was trained using concentration-averaged, single-species spectra of the constituents of the mixture as training vectors and their prepared nominal concentration as target vectors. The three components, i.e., LVs, of the model correspond to the known chemical dimensionality of the training set.

A multi-species PLS model was then developed using all individual spectra with concentrations exceeding the corresponding LOQ values as training set. Concentration-averaged spectra of pure analytes were used as training inputs, with their prepared nominal concentrations as target values. The model employed three latent variables, corresponding to the known chemical dimensionality of the system. The model was cross-validated via a LOGO^62^ procedure, used to retrieve the concentrations of individual species in the ternary mixtures. This was implemented via the Python machine learning library scikit-learn (v1.8.0)^63^. The LOGO-CV held out one concentration level at each fold. Such grouping scheme prevents data leakage and provides an unbiased assessment of predictive performance. The analysis of the cross-validation error showed that three LVs captured more than 99% of the variance in the training dataset, yielding a root-mean-squared error of cross-validation of 1.4 ppm. In addition, the model performance was evaluated using a binary acetone-pentane mixture as an external validation set. The corresponding root mean squared errors of prediction were 2.5, 5.4 and 7.5 ppm for methanol, acetone and pentane, respectively. Results of validation with external data sets for binary mixtures are presented in Table S2 in the supplementary information.

## Results and discussion

**Fig. 2:**
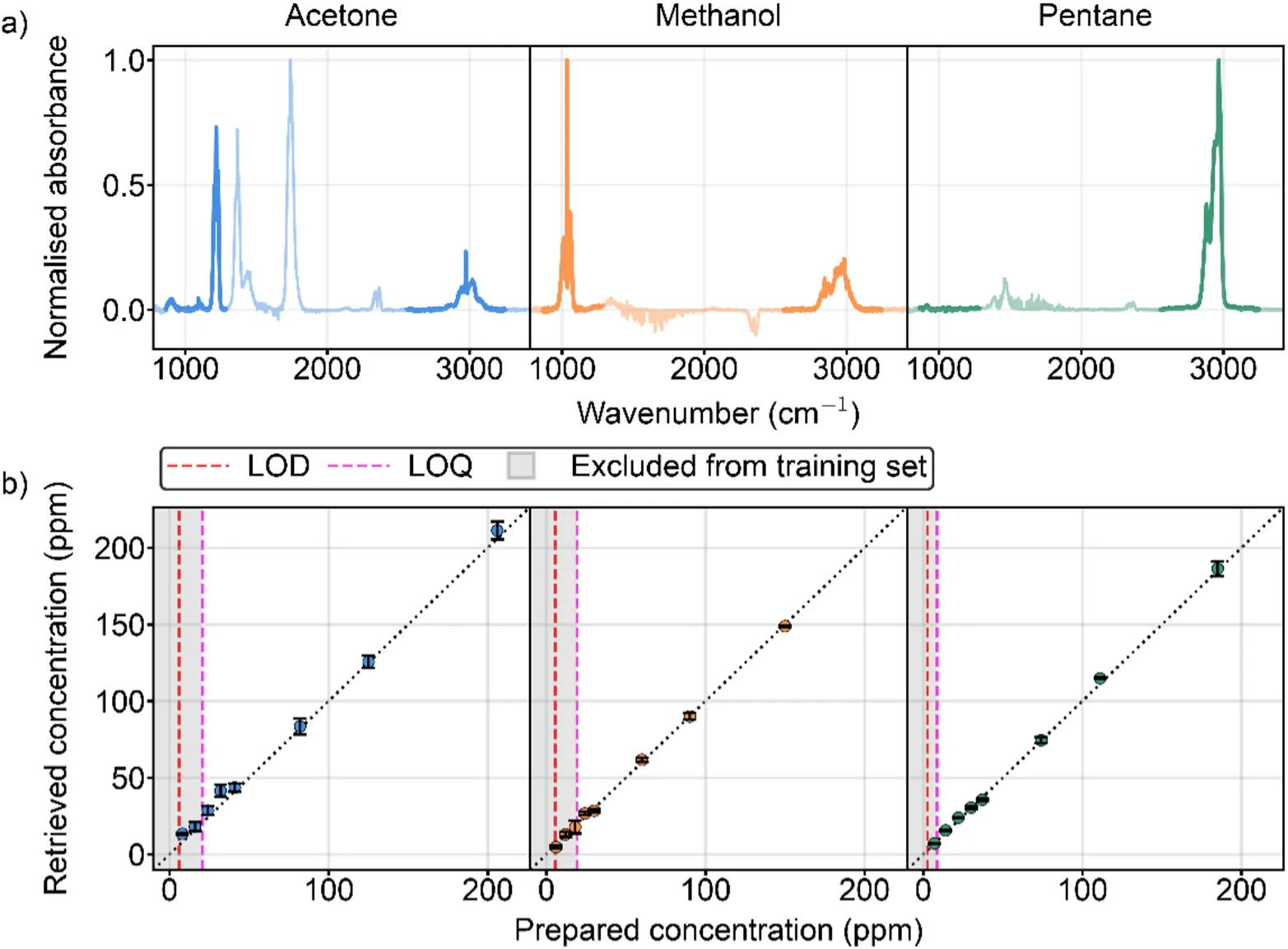
Broadband IR vibrational spectroscopy analysis of gaseous acetone (left), methanol (middle) and pentane (right) diluted in nitrogen. a) Absorbance spectra normalised to their respective maxima. The spectral regions indicated by the darker lines were used for PLSR-based concentration retrieval. The spectra in (**a**) correspond to the highest prepared concentration. (**b**) Average cross-validation concentration predictions as a function of prepared concentrations. The error bars correspond to +/- standard deviation of retrieved concentrations for the replicate samples at identical concentrations. The dotted grey line indicates ideal agreement (retrieved concentration equals the prepared one). The dotted red line indicates the LOD, while the pink indicates the LOQ. The shaded grey region indicates concentration ranges excluded from the training set of the multi-species PLS model.

Gas mixtures of acetone, methanol, and pentane were used to demonstrate the use of the gas sampling unit for real-time spectroscopic monitoring of gas from a ventilated artificial test lung. First, gaseous samples containing different concentrations ranging from several ppm up to a few hundred ppm containing only a single selected compound diluted in nitrogen were prepared (see Table S2 in the supplementary information), measured, and analysed. The samples were prepared without connection to a respirator (Figure S1 in the supplementary information). Figure 2 shows the characteristic infrared absorbance spectra of the three selected species as well as the concentration retrieval based on partial least squares regression (PLSR) models.

All three studied species exhibit distinct and pronounced absorption features in the mid-IR spectral region. For acetone, these can be observed in the ranges 1180–1250, 1330–1400, 1690–1790, and 2900–3050 cm^− 1^. For methanol, the most prominent features occur in the ranges 960–1080 and 2740–3000 cm^− 1^. Pentane shows pronounced features in the 1830–1300 cm^− 1^, as well as in the ranges 1350–1500 and 840–1030 cm^− 1^. For the PLSR-based concentration retrieval, the spectral regions 865–1285  and 2560–3450 cm^− 1^ were used. The spectral region between these bands (i.e., lighter curve region in Fig. 2a) was excluded due to interference from water vapour and carbon dioxide, which can significantly affect retrieval accuracy, particularly at low analyte concentrations. Acetone and pentane were selected due to their established relevance in clinical studies, in particular for metabolic monitoring in breath (e.g. ketosis, oxidative stress). Methanol was chosen due to its richness in the spectral profile, partial overlapping of bands with the other chosen gases – which will happen in real-breath analysis – as well for reasons of sensitivity comparison with state-of-the-art field-resolved spectroscopy^55^.

Figure 2b shows the result of the PLSR-based calibration model for the three studied species. Separate PLSR models were trained for each species, and the concentrations were retrieved, showing good agreement with the prepared values. Minor deviations from the expected values can be attributed to the uncertainties in sample preparation as well as measurement uncertainty. From the dilution series, LOD and LOQ values for all three studied species were determined and are summarised in Table 1.

**Table 1:** LOD and LOQ for acetone, methanol, and pentane.

	Acetone	Methanol	Pentane
LOD (ppm)	6.03	5.61	2.5
LOQ (ppm)	20.6	19.2	8.6

To demonstrate the applicability of the newly developed gas sampling unit for continuous gas sampling from a respirator (Fig. 1), the studied species were introduced into the airflow of the ventilated test lung. This was performed by connecting a reservoir containing a liquid mixture of the studied species via a short tube with a manual valve (see Materials and methods and Fig. 1). Vapour from the liquid phase diffuses into the ventilated pathways upon valve opening (Manual valve, Fig. 1).

**Fig. 3:**
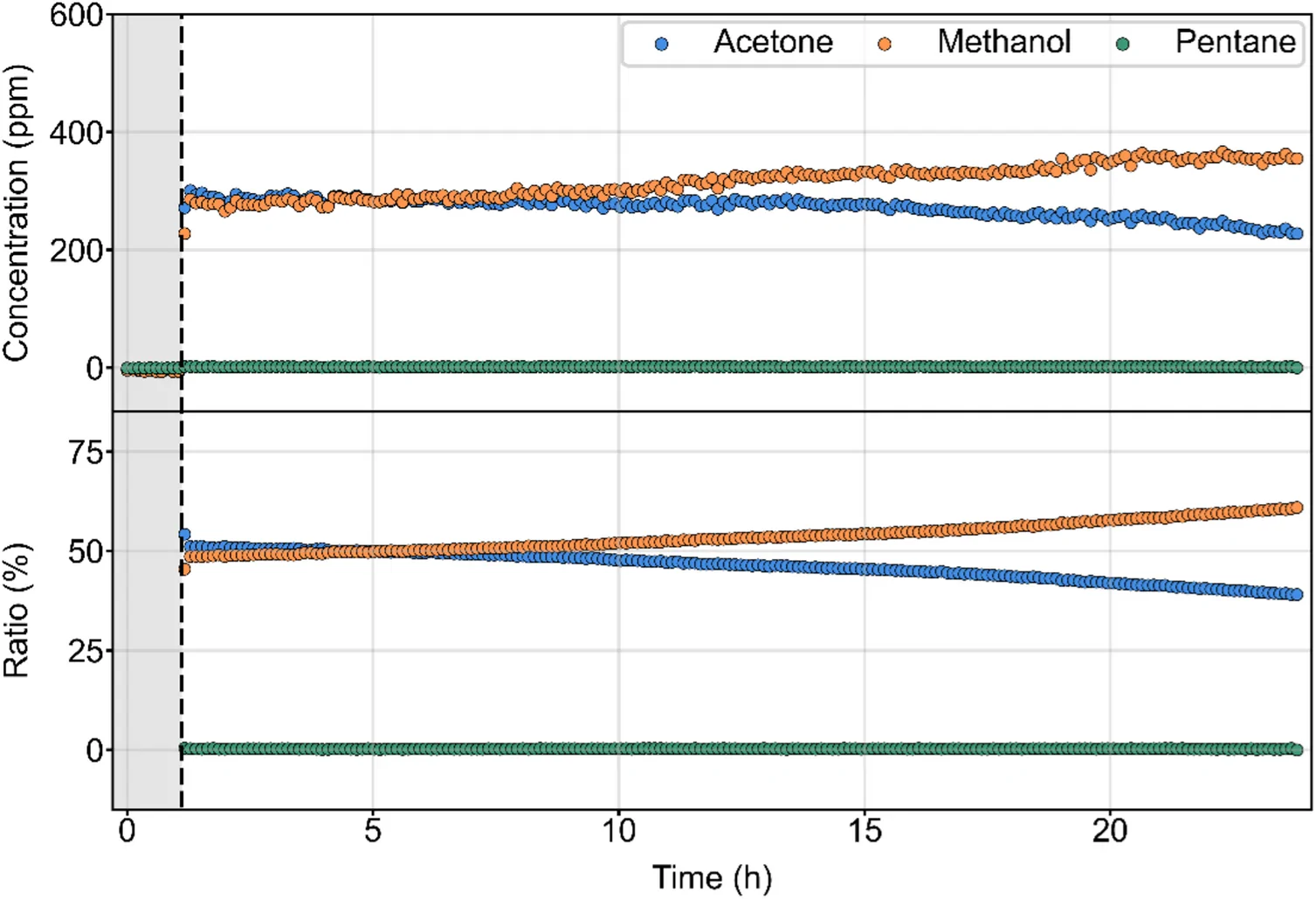
Continuous, 24-hour monitoring of pentane, methanol and acetone concentrations in the airflow of the mechanically ventilated test lungs resulting from the liquid binary mixture (acetone-methanol). Upper panel: retrieved concentration. Lower panel: concentration ratio of monitored species calculated as the concentration of each individual species divided by the sum of all three species. Note that the valve allowing vapour of the studied species to enter the ventilated pathways was open one hour after the measurement started (vertical black dashed line).

For concentration retrieval during continuous gas sampling experiments, a dedicated multi-species PLSR model was used (see Materials and methods section) to simultaneously determine concentrations of all three studied species (acetone, methanol and pentane) from each measured absorbance spectrum. The result of a 24-hour continuous measurement of a binary mixture (acetone-methanol) is shown in Fig. 3.

After an hour of gas sampling from the respirator and subsequent spectroscopic measurements, the valve (directly at the sample bulb) allowing vapour of the binary mixture to enter the ventilated pathways was opened. Consequently, a sudden rise in the concentrations of acetone and methanol can be observed, while the concentration of pentane remained zero, as expected. Initially, the concentrations of acetone and methanol exhibit similar values (~ 280 ppm) with acetone having slightly higher concentration. Over time, the methanol concentration starts increasing and exceeds the concentration of acetone, whose concentration is gradually decreasing.

These trends (the retrieved concentrations and concentration ratios) are directly related to the vapour pressure of the studied species in the sample bulb connected to the ventilated pathways. The vapour pressures of mixed species are proportional to mole fractions and pure-component vapour pressure. Although the mixture was prepared equivolumetrically, methanol exhibits a higher mole fraction (65%), whereas acetone (35% mole fraction) has a higher vapour pressure^64^. This leads to the two mixing components initially having similar vapour pressure, which is observed as similar retrieved concentration from the ventilated pathways in the initial phase of the experiment.

As the measurement evaporation proceeds further, acetone vapour and methanol vapour continue diffusing from the sample bulb into the ventilated pathways and the test lungs. The species diffused from the sample bulb are replaced by molecules evaporating from the liquid mixture. This in turn reduces the number of molecules in liquid form in the reservoir. In the beginning, the two compounds have a comparable vapour pressure, which means that a comparable number of molecules diffuse into the ventilated pathways and evaporates from the liquid phase. However, due to the smaller number of acetone molecules in the initial mixture, the mole fraction of acetone decreases even further, which consequently reduces the partial pressure of acetone and therefore the concentration of acetone in the ventilated pathways. In the binary mixture the mole fraction of methanol increases at the same time as a direct consequence of the decreased acetone mole fraction. Consequently, the concentration of methanol increases with time as observed in the experiment. After 24 h the experiment was terminated, with liquid still visibly present in the sample bulb, which is consistent with the observed concentrations of methanol and acetone in the final phase of the experiment.

**Fig. 4:**
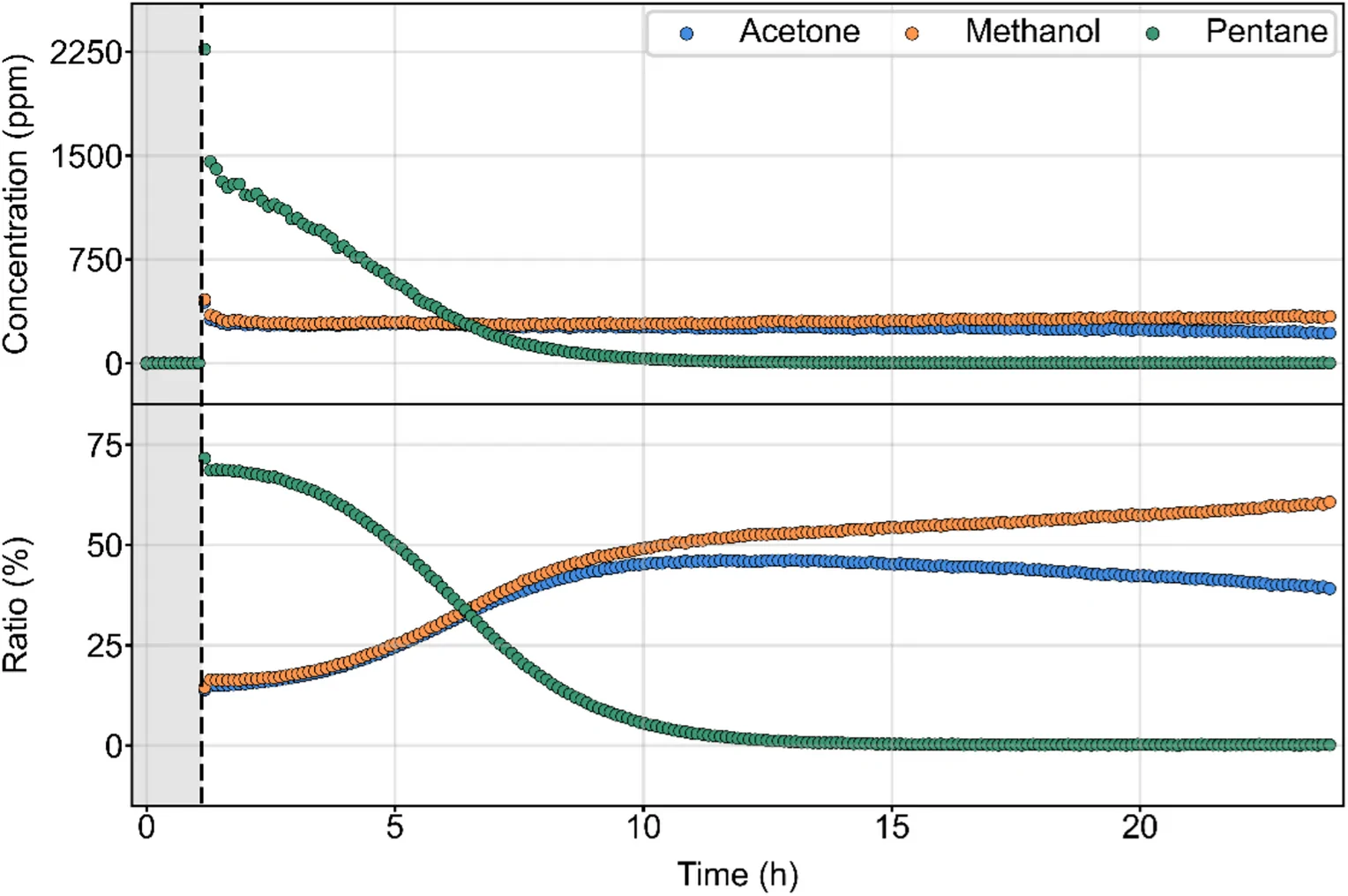
Continuous, 24-hour monitoring of pentane, methanol and acetone concentrations in the airflow of mechanically ventilated test lungs resulting from the liquid ternary mixture (acetone-methanol-pentane). Upper panel: retrieved concentration. Lower panel: concentration ratio of monitored species calculated as the concentration of each individual species divided by the sum of all three species. Note that the valve allowing vapour of the studied species to enter the ventilated pathways was opened one hour after the measurement started (vertical black dashed line).

In a second 24-hour measurement, the experiment was modified by additionally introducing pentane into the mixture placed in sample bulb (see Materials and methods section). The results of the second 24-hour measurement or ternary mixture are shown in Fig. 4.

After the valve opening allowing the vapour of the ternary mixture to enter the ventilated pathways, the dynamics are initially dominated by pentane, which has the highest vapour pressure of the three investigated species. Furthermore, pentane is non-miscible with methanol^65^ and only poorly miscible with acetone^66^. This makes the interactions of the three species in the liquid mixture and the corresponding dynamics more complex. Nevertheless, tracking the concentration of the three species in the sampled gas reveals that the concentration of pentane decreases comparably fast despite being substantially higher in the beginning of the experiment. This can be attributed to the high vapour pressure of pentane. After approximately 12 h, the concentration of pentane approaches zero indicating its complete evaporation. After this point, the concentration trends of acetone and methanol as well as their ratios match those from the first 24-hour experiment (see Fig. 3). Methanol concentrations are overall higher than those of acetone, likely due to the reduced acetone vapour pressure caused by interactions with pentane within the mixture. Furthermore, the vapour pressures are temperature dependent, so that different room temperatures during the two experiments could contribute to the observed differences between the two 24-hour experiments.

The observed dynamics of the retrieved concentrations in both experiments are reflected more clearly in the relative concentration ratios, which provide more stable metrics, less prone to fluctuations caused by temperature variations and system instabilities. The ratio for each studied species was calculated as the quotient of its concentration and the total concentration of all three monitored species. For clinical applications, distinguishing physiologically relevant VOC changes from environmental or instrumental fluctuations is essential. Monitoring ratios of multiple VOC species, which draws from the inherently multi-variate nature of broadband spectroscopy, provides a robust strategy to achieve this.

Breath is a complex mixture containing many different molecules. On one hand, medically relevant VOCs are typically present at much lower physiological concentrations (ppb or ppt levels) than those measured in this study. Consequently, significantly better detection sensitivity is required for real-life applications compared to the achieved LOD values reported in this study. One way to improve the sensitivity is to increase the interaction path length of IR radiation with the gas sample, for example by employing a multi-pass gas cell. The sensitivity can be further improved by employing more advanced, laser-based broadband spectroscopy techniques such as IR field-resolved spectroscopy (FRS), which has been shown to detect e.g., methanol at concentrations up to three orders of magnitude lower than those reported here, even when using a single-pass gas cell of similar length^55^. Additional improvements might be achieved by investigating breath samples in enhancement cavities in combination with FRS^67^. On the other hand, breath also contains VOCs that are present at much higher concentrations compared to the relevant biomarkers. Water and carbon dioxide are two common examples that can complicate the detection of more relevant VOCs due to overlapping spectral features. The effects of spectral overlap can be reduced by conducting the measurements at lower pressures and sufficiently high spectral resolution^68^. Furthermore, techniques such as modulated ringdown comb interferometry^56^ have been shown to mitigate this problem. Such modifications, as well as the investigation of real breath samples containing physiological levels of carbon dioxide and water vapour need to be further validated for clinical applications.

Within the 24-hour experiments, the gas from the respirator was spectroscopically analysed every seven minutes. The duration of the measurement cycle depends on the time required for the evacuation and filling of the measurement cell, and the time required to measure both reference sand sample spectra (~ 5 min). For the two demonstrated experiments the temporal resolution was mainly defined by the duration of the FTIR measurements and could be improved with faster spectroscopic investigation (i.e., reduced number of scans), however, at a cost of worse sensitivity. The time required for the evacuation and filling of the measurement cell depends on the desired vacuum levels, employed vacuum pump, valves and connecting tubes, the measurement cell volume as well as gas sampling flow rate and the selected sample pressure. The selected target pressure of 1.5 mbar in these experiments was achievable within a few tens of seconds. To achieve substantially lower pressures, the employed tubes, valves and measurement cell should be exchanged with corresponding high-vacuum components.

Evacuation of the measurement cell is a crucial step for performing the reference measurement and exchanging the sample. The lower the achievable pressure, the less molecules from the previous sample measurement and from the surrounding (due to potential leakages) can influence the measurements. Furthermore, flushing the measurement cell with nitrogen gas of high purity, in combination with evacuation can improve cleaning of the cell and can be easily introduced within the current gas sampling unit. To further reduce potential contamination, heating the measurement cell during evacuation and nitrogen flushing could be applied, accelerating the desorption of molecules from the measurement cell walls. Introducing a turbo pump to the setup would help to reduce the pressure and sample carryover even further. However, such modification come at a cost of higher system complexity and higher cost. Furthermore, such extensions can substantially influence the temporal resolution of the measurements. The optimal balance between these factors depends on the intended clinical application.

Overall, the presented measurements demonstrate the feasibility of broadband optical spectroscopy in combination with continuous breath sampling from mechanically ventilated patients over extended periods, ranging from hours to days. This capability highlights the potential of such systems for real-time monitoring of patient status, which is particularly relevant for conditions such as sepsis, where rapid physiological changes necessitate continuous observation.

## Conclusion and outlook

In this proof-of-principle study, we have introduced, for the first time, a fully integrated and automated system that enables direct and continuous monitoring of breath from mechanically ventilated airways with broadband optical spectroscopy. The developed approach eliminates the need for intermediate sample storage, allowing for real-time monitoring of exhaled gases. The experiments, conducted with FTIR spectroscopy of a simple matrix only using chosen synthetic gases in a ventilated test lung, highlight that the presented configuration does not interfere with respirator operation at any stage. Moreover, the ability to perform uninterrupted measurements over extended periods (24 h and beyond) confirms the stability and suitability of the system for long-term monitoring applications.

With the PLRS-based concentration retrieval we showcase the ability of broadband optical spectroscopy to detect and monitor the concentration of multiple VOCs simultaneously. This in turn enables tracking concentration ratios of VOCs and their changes that are less susceptible to systematic fluctuations than the concentrations itself and therefore provide a more reliable way for identification of physiologically relevant changes. With the employed FTIR system and measurement cell, the limits of detection and quantification for the studied VOCs lie in the ppm range, which is above the typical physiologically relevant concentrations. Accordingly, enhancing detection sensitivity, along with spectral resolution remain key objectives to reach physiologically relevant concentrations of VOCs present in human breath. One way to improve the sensitivity is by implementation of a multi-pass gas cell or optical enhancement cavity, which significantly extends the optical path length, thereby improving signal intensity and the detection limits. In parallel, the integration of advanced spectroscopic techniques such as FRS and ringdown spectroscopy offers the potential for orders-of-magnitude sensitivity improvement, enabling detection of trace-level biomarkers also in presence of more abundant volatiles. Further hurdles arise from intricate gas mixtures as true exhaled air. As such, the applicability of the improved method for breath studies needs to be properly validated on physiologically relevant samples that properly resemble breath in its entire complexity.

These advancements, combined with further refinement of chemometric models, will bring the proposed system closer to real-world clinical applications. An important strength of our approach is the adaptation of our spectroscopy device via approved sampling technologies (Vamos) to the clinical respirator enabling regulatory approval of such strategies in a real-world intensive care unit (ICU) setting. Ultimately, this lays the groundwork for continuous, non-invasive monitoring of breath biomarkers in mechanically ventilated patients with potential implications for early diagnosis and therapy monitoring in the context of personalized medicine for the critically ill.

## Supplementary Information

Below is the link to the electronic supplementary material.

Supplementary Material 1

## Data Availability

The data generated during the current study will be made available from the corresponding author on a reasonable request.
