# Supplementary material for: Respirator-integrated continuous gas sampling for broadband optical spectroscopy
Source: Sci Rep. 2026 Sep 4;16:27809. doi: 10.1038/s41598-026-69327-3 (PMC13545246; doi:10.1038/s41598-026-69327-3)
Supplement: Supplementary file 1 [file 41598_2026_69327_MOESM1_ESM.docx]

SUPPLEMENTARY INFORMATION

**Respirator-integrated continuous gas sampling for broadband optical spectroscopy**

Aleksandra Borek-Dorosz^1,2†^, Jan Ornik^1,2†^, Marco Personeni^3^, Stefan Haering^3^, Maximilian Högner^3^, Felix Paries^3,4^, Margit Leitner^5^, Jens-Uwe Weiss^6^, Ignacio Rubio^5,6^, Michael Bauer^2,5,6^, Ioachim Pupeza^1,2,3,4^*

^1^Leibniz Institute of Photonic Technology ─ Member of the Research Alliance, Leibniz Health Technologies, 07745 Jena, Germany

^2^Cluster of Excellence Balance of the Microverse, Friedrich Schiller University Jena, 07743 Jena, Germany

^3^Physics Department and State Research Center OPTIMAS, University of Kaiserslautern-Landau, 67663 Kaiserslautern, Germany

^4^Fraunhofer Institute for Industrial Mathematics ITWM, 67663 Kaiserslautern, Germany

^5^Department of Anesthesiology and Intensive Care, member of the Leibniz Center for Photonics in Infection Research (LPI), Jena University Hospital, Friedrich Schiller University, Am Klinikum 1, 07749 Jena, Germany

^6^Center for Sepsis Control and Care (CSCC), Jena University Hospital, Am Klinikum 1, 07749 Jena, Germany

* Corresponding author: ioachim.pupeza@rptu.de

^†^ Contributed equally

**This material includes:**

M.1. Cleaning procedures of the glass bulbs and gas-tight syringes.

Figure S1. Schematic representation of preparation setup.

M.2. Concentration calculations equations.

M3. Detailed respirator settings used in experiments.

Table S1. List of prepared single species for FTIR measurements.

Table S2. PLS external validation.

M.1. Cleaning procedures of the glass bulbs and gas-tight syringes

*Glass bulb cleaning procedure*

At first, all the parts of the preparation setup we evacuated using a vacuum pump. To speed up the cleaning process, all valves were closed, except those directly connected to the glass bulb (i.e., stock or sample bulb.,V3, V6/V7, Vb1/Vb3), and evacuation was continued for 2 minutes. In the next step, the vacuum pump was turned off, and all valves were closed. The flushing step involved allowing dry air, and subsequently nitrogen, to pass through the entire sample bulb (both valves on the glass bulb were opened). The second valve on the bulb (Vb2/Vb4, Figure S1), the one not connected to the preparation setup, was left open to the room. The flushing process took around 5 minutes. The evacuation and flushing with air were repeated 2–4 times (for higher concentrations, even more repetitions were required).

After working with different species, the bulb was cleaned using soap and a cleaning kit (Hamiltonian cleaning kit). First, the glass bulb was disconnected from the preparation system, and all parts were disassembled (valves and septum removed). The bulb was initially rinsed with warm water, then cleaned with warm water mixed with soap, and finally with dishwasher liquid. The soap and dishwasher liquid were left inside the bulb to soak for around 30 minutes. Residues of the cleaning products were removed by flushing the bulb several times with warm water. In the next step, the bulb was treated with diluted Hamilton solution; a few millilitres were added to the bulb and mixed with warm water. To ensure better detachment of molecules from the measurement cell walls, the bulb containing the cleaning solution was rotated several times. The remaining solution was then rinsed out with water several times. All parts were reassembled (except for the septum). During rising the bulb with water, and didn`t see foam formation, the bulb was connected to a flow of dry air, and the flushing procedure was carried out for around 10–15 minutes. Once all water droplets had been removed, the septum was reinstalled, and the bulb was reconnected to the preparation setup. Finally, the evacuation process was initiated, followed by flushing with dry air or nitrogen and subsequent evacuation; these steps were repeated several times. To ensure that the bulb was free of contamination, FTIR spectra were recorded (reference: empty FTIR measurement compartment).

*Gas-tight syringe cleaning procedure*

First, the syringes used exclusively for transferring gas samples were cleaned using the preparation system. The syringe was inserted through the septum into the glass bulb. Initially, evacuation was carried out, and the piston was raised several times. Next, the piston was removed from the syringe in the same configuration, and the flushing procedure was performed. The evacuation and flushing processes were repeated at least three times.

In the case of syringes used for liquid samples, the cleaning procedure required additional steps. First, all parts of the syringe were disassembled (needle, piston, etc.). All components were initially soaked in warm water for a few minutes and then in a Hamilton cleaning solution for approximately 30 minutes. After the required time, all parts were thoroughly rinsed with water and left to air-dry. Subsequently, all parts were reassembled, and the flushing and evacuation procedures were performed exactly as described for syringes used for gaseous samples.

There are several sources of uncertainty that affect the concentration accuracy of the prepared gas samples. For the syringes, the volume accuracy is on the order of ±1% as specified by the producer. The temperature fluctuations were below 2%, and ambient pressure fluctuations are typically below 5%. Considering the relations between the quantities in equations S1-S5, the uncertainty of the sample preparation uncertainty is approximately 10%.

Figure S1. Schematic representation of preparation setup**.** Preparation setup of samples for calibration curves, with defined concentrations, which consists of measurement cell located in FTIR spectrometer, and its connection to the pressure sensor, vacuum pump and nitrogen bottle. V – valves, Vb – bulb valves.

M.2. Concentration calculations equations

$n_{s}=V_{s}\cdot\frac{\rho}{M}$	*Equation S1*

$p_{s =\frac{n_{s}\cdot R\cdot T}{V_{b}}}$	*Equation S2*

$C_{s}=\frac{p_{s}}{p_{0}}\cdot{10}^{6}$	*Equation S3*

$D=\frac{V_{b}}{V_{d}}$	*Equation S4*

$C_{d}=\frac{C_{s}}{D}$	*Equation S5*

Where:

$C_{s}$ – concentration of stock [ppm]

$C_{d}$ – concentration of diluted samples [ppm]

$p_{s}$ – partial pressure of the substance [Pa]

$p_{0}$ – standard atmospheric pressure 100 000 [Pa]

$n_{s}$ – amount of substance [mol]

$R$ – gas constant (8.31 10^6^ [(cm^3^·Pa)/(K·mol)])
$T$ – temperature [K] (assumed 293 K for calculations)
$V_{b}$ – volume of bulb [cm^3^]

$V_{s}$ – volume of sample injected into bulb [cm^3^]

$V_{d}$ – volume of gaseous sample transferred from the stock into bulb [cm^3^]

$\rho$ – density [g/cm^3^]

$M$ – molar mas [g/mol]

$D$ – dilution factor

M3. Detailed respirator settings used in the experiments.

The respirator was set with the following settings: 21% of oxygen, 500 cm^3^ breathing volume (VT), AutoFlow (AF) equal 10, Positive End Expiratory Pressure (PEEP) 5.0, Time (Ti) 2.0, Support Pressure (Psupp) 5. To ensure real conditions, the respirator was connected to oxygen and air, provided in every patient room via the internal hospital gas supply system.

Table S1. List of prepared single species for FTIR spectroscopy measurements. The concentrations used to generate the dilution series for acetone, pentane, and methanol are summarized. The blue-highlighted section indicates the preparation of stock solutions in a 1,000-cm³ bulb, specifying the concentration (ppm) and the volume (μl) of liquid sample used to achieve it. Subsequent rows show the various concentrations prepared for each species to prepare diluted samples, detailing the final concentration (in ppm, in 1,000-cm^3^ bulb), and the volume (μl) transferred from the stock solution for each target concentration.

|  | **Acetone** | | **Pentane** | | **Methanol** | |
| --- | --- | --- | --- | --- | --- | --- |
| **Stock** | **Concentration (ppm)** | **Liquid volume (μl)** | **Concentration (ppm)** | **Liquid volume (μl)** | **Concentration (ppm)** | **Liquid volume (μl)** |
|  | **82,264** | **250** | **73,978** | **350** | **60,294** | **100** |
| **Diluted samples** | **Concentration (ppm)** | **Volume (μl) from stock** | **Concentration (ppm)** | **Volume (μl) from stock** | **Concentration (ppm)** | **Volume (μl) from stock** |
| S1 | 8 | 100 | 7 | 100 | 6 | 100 |
| S2 | 16 | 200 | 14 | 200 | 12 | 200 |
| S3 | 24 | 300 | 22 | 300 | 18 | 300 |
| S4 | 32 | 400 | 30 | 400 | 24 | 400 |
| S5 | 41 | 500 | 37 | 500 | 30 | 500 |
| S6 | 82 | 1,000 | 74 | 1,000 | 60 | 1,000 |
| S7 | 125 | 1,500 | 111 | 1,500 | 90 | 1,500 |
| S8 | 206 | 2,000 | 185 | 2,500 | 150 | 2,500 |

Table S2. PLS external validation. Nominal concentrations of the prepared mixtures and the concentrations predicted by the three-component PLS model. External validation samples were prepared using the gas generation system without preconcentration on the respirator. Concentrations are expressed in ppm and reported as the mean of three independent measurements for each concentration level.

| Prepared concentration (ppm) | | | Retrieved concentration (ppm) | | |
| --- | --- | --- | --- | --- | --- |
| Acetone | Pentane | Methanol | Acetone | Pentane | Methanol |
| 125 | 185 | 0 | 124,4 | 197,1 | 3,4 |
| 16 | 185 | 0 | 15,1 | 196,5 | 1,4 |
| 206 | 14 | 0 | 207,3 | 16,4 | -0,4 |
| 24 | 111 | 0 | 21,0 | 116,4 | -0,1 |
| 24 | 30 | 0 | 19,6 | 31,1 | 1,8 |
| 32 | 37 | 0 | 24,7 | 37,3 | -2,1 |
| 41 | 74 | 0 | 39,5 | 75,8 | 0,2 |
| 8 | 30 | 0 | 13,6 | 47,8 | -2,4 |
| 82 | 22 | 0 | 75,5 | 25,4 | -2,1 |
| 82 | 37 | 0 | 81,8 | 36,2 | 0,1 |
